# Clinical practice in using corticosteroids and adjunctive sepsis therapies at the bedside among European ICUs: an ESICM-endorsed survey

**DOI:** 10.1186/s40635-026-00877-6

**Published:** 2026-03-10

**Authors:** Sascha David, Marc Leone, Massimo Girardis, Mattia M. Müller, Srdjan Gavrilovic, Roberta Domizi, Elisa Damiani, Ignacio Martin-Loeches, Ricard Ferrer, Benjamin Chousterman, Lene Russell

**Affiliations:** 1 Institute of Intensive Care Medicine, University Hospital Zurich & University of Zurich, Rämistrasse 100, CH-8032 Zurich, Switzerland; 2 Department of Nephrology, Medical School Hannover, Hannover, Germany; 3 Department of Anesthesiology and Intensive Care Medicine, Nord Hospital, Assistance Publique Hôpitaux Universitaires de Marseille, Aix Marseille University, Marseille, France; 4 Department of Anesthesia and ICU, University Hospital of Modena, Modena, Italy; 5 Faculty of Medicine, University of Novi Sad, Novi Sad, Serbia; 6 Institute for Pulmonary Diseases of Vojvodina, Sremska Kamenica, Serbia; 7 Clinic of Anesthesia and Intensive Care, Department of Biomedical Sciences and Public Health, Università Politecnica Delle Marche, Ancona, ItalyClinic of Anesthesia and Intensive Care, Azienda Ospedaliero Universitaria Delle Marche, Torrette, Italy; 8 Department of Intensive Care Medicine, Multidisciplinary Intensive Care Research Organization (MICRO), St James’s Hospital, Dublin, D08 NHY1 Ireland; 9 School of Medicine, Trinity College Dublin, Dublin, D02 PN40 Ireland; 10 Trinity Centre for Biomedical Engineering, Dublin, Ireland; 11 Intensive Care Department, Vall d’Hebron Hospital Universitari, SODIR Research Group, Vall d’Hebron Institut de Recerca (VHIR), Department of Medicine, Universitat Autònoma de Barcelona. Vall d’Hebron Barcelona Hospital Campus, Passeig Vall d‘Hebron 119-129, 08035 Barcelona, Spain; 12 Université Paris Cité, Paris, France; 13 Department of Anesthesia and Critical Care, AP-HP, Hôpital Lariboisière, Paris, France; 14 Department of Intensive Care, Herlev-Gentofte Hospital, Copenhagen, Denmark; 15 Department of Clinical Medicine, Copenhagen University, Copenhagen, Denmark

**Keywords:** Shock, Blood purification, Steroids, Immunoglobulin, Immunomodulation

## Abstract

**Background:**

Sepsis and septic shock remain major causes of morbidity and mortality worldwide, and management is largely based on source control, antimicrobial therapy, and supportive care. Despite limited high-quality evidence, adjunctive therapies targeting the dysregulated host response involving immune dysfunction, coagulopathy, and endothelial injury, steroids, vasopressors, and adjunctive therapies are frequently used in clinical practice. This survey aimed to describe real-world patterns of sepsis therapy across Europe.

**Methods:**

We conducted an open, web-based, multinational survey endorsed by the European Society of Intensive Care Medicine (ESICM) and the Italian Society of Anesthesia, Analgesia, Resuscitation and Intensive Care (SIAARTI). The survey was distributed through professional mailing lists, newsletters, and national society networks. A structured 30-item questionnaire collected information on respondent demographics, ICU characteristics, availability of adjunctive therapies, clinical indications, triggers for initiation, and duration of treatment. Participation was voluntary and anonymous. A total of 442 physicians completed the survey. Data were analyzed descriptively and are presented as proportions and frequencies.

**Results:**

More than 80% of respondents reported use of at least one adjunctive therapy for septic shock within the previous year. Corticosteroids were used by over 90% of clinicians, predominantly hydrocortisone for septic shock. Considerable variability was observed regarding indications, timing of initiation, and duration of therapy. Extracorporeal blood purification techniques were used by approximately 75% of respondents, most frequently hemoadsorption in patients with refractory shock; high cost and limited availability were the main barriers to broader implementation. Intravenous immunoglobulins were used by approximately one-third of clinicians, often guided by measured immunoglobulin levels or perceived immune dysfunction. Additional vasoactive and inotropic agents, including levosimendan, methylene blue, and beta-blockers, were employed in selected cases. In contrast, specific immunomodulatory therapies such as interleukin (IL)-1receptor antibodies were rarely used. Across all adjunctive strategies, marked heterogeneity in practice patterns was evident.

**Conclusion:**

Adjunctive therapies are widely used in European ICUs, particularly in patients with severe or refractory sepsis, despite limited supporting evidence. The substantial variability in practice highlights ongoing clinical uncertainty and underscores the need for well-designed randomized trials to inform evidence-based and individualized treatment strategies.

## Introduction

Sepsis is a major health care problem affecting over 50 million people annually worldwide leading to an estimated death of 11 million [1]. Sepsis is not a disease per se but rather a syndrome defined as life-threatening organ dysfunction due to a pathological host response to an infection [2]. Its most severe form known as septic shock has a mortality rate up to 40% even in modern intensive care units (ICUs). Yet, the treatment is limited to source control, antimicrobials, and supportive measures such as volume resuscitation, vasopressors, and organ support including mechanical ventilation and kidney replacement therapy [1, 3, 4]. Given the central role of the pathological host response, one might wonder if interventions focusing on this self-injurious response of the organisms might be causative and thus more effective [5]. The host response includes alterations in three areas, i.e., the immune response, coagulopathy, and endothelial dysfunction [6]. In this manuscript, we use the term adjunctive therapies to refer to treatments with a certain biological plausibility to target the injurious host response. The field of these interventions is wide [7] and ranges from anticoagulants [8] to immunosuppressants/boosters [9] and even extracorporeal strategies [10, 11].

Despite decades of research, sepsis outcomes remain unacceptably poor, and no host-directed adjunctive therapy has yet demonstrated consistent benefit across heterogeneous patient populations. Nevertheless, such therapies continue to be widely used in clinical practice, often driven by biological plausibility, pathophysiological reasoning, and local experience rather than robust evidence. We hypothesized that the use of adjunctive therapies in sepsis varies substantially across regions and clinical contexts, reflecting uncertainty in patient selection, timing, and stopping rules. To test this hypothesis, we conducted an international survey of intensivists to characterize real-world practice patterns, including epidemiology, indications, stratification strategies, and regional preferences for steroids, vasopressors, and adjunctive therapies. This work is particularly timely given the increasing emphasis on precision medicine in sepsis, the emergence of novel immunomodulatory and extracorporeal approaches, and renewed interest in targeting host response pathways. Understanding how these therapies are currently applied in routine care is a critical step toward informing future trials, harmonizing practice, and identifying areas of genuine clinical equipoise.

## Methods

### Design

This study was conducted as an open, web-based, multinational survey designed to explore real-world clinical practice related to adjunctive therapies in sepsis. The survey methodology was chosen to capture contemporary practice patterns across different health care systems and geographical regions.

### Endorsement and dissemination

The survey was formally endorsed by the European Society of Intensive Care Medicine (ESICM). It was hosted on the ESICM website and promoted through the ESICM Newsletter. In addition, dissemination was supported through the professional networks of the co-authors and via the Italian Society of Anesthesia, Analgesia, Resuscitation and Intensive Care (SIAARTI). Participation was voluntary and not restricted to ESICM or SIAARTI members.

### Participants

**Fig. 1:**
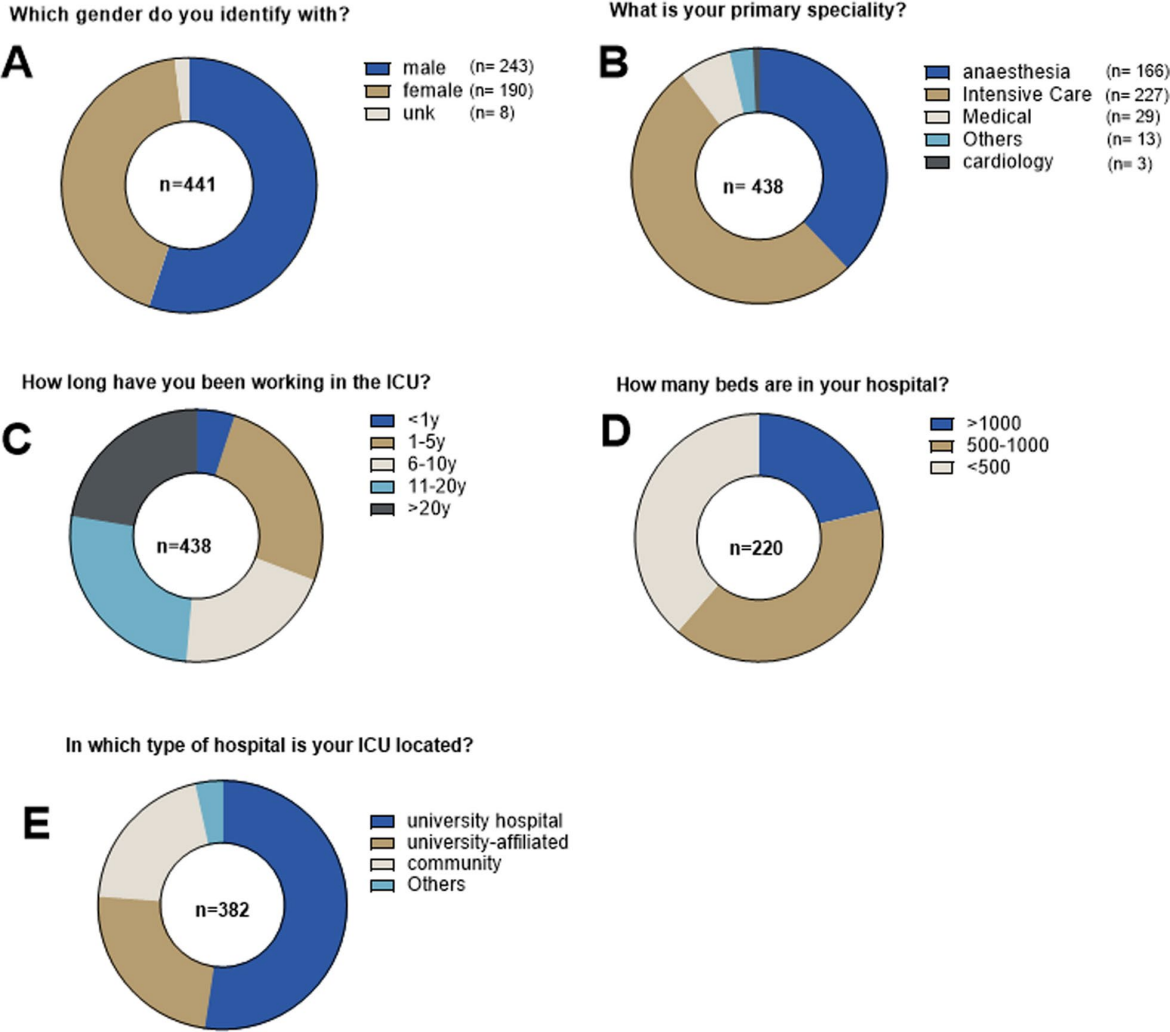
Characteristics of participants

The target population consisted primarily of intensivists involved in the management of patients with sepsis. However, participation was not limited by subspecialty or level of training, allowing inclusion of respondents with heterogeneous professional backgrounds and clinical experience (Fig. 1). A total of 442 participants completed the survey.

### Survey instrument

The survey consisted of 30 consecutive questions, all of which were completed anonymously. Questions were primarily multiple-choice, requiring respondents to select one answer from a set of predefined options. For a subset of questions, respondents were given the opportunity to provide user-defined additional responses to capture practices not covered by the predefined choices (Table 1).

**Table 1:** Survey questions

Q No.	Topic/question
	Demographics
1	Which gender do you identify with? (one option)
2	What is your primary speciality? (one option)
3	In which TYPE of hospital is your ICU located? (multiple options possible)
4	How many beds are in your hospital
5	How long have you been working in the Intensive Care Unit? (one option)
6	In which country do you practice?
	Corticosteroids
7	Have you used STEROIDS in patients with sepsis/septic shock within the last year? (multiple options possible)
7a	Why are you not using steroids?
8	Which steroids do you prefer in septic shock patients? (multiple options)
9	What is your clinical trigger to initiate steroids in a septic shock patient? (multiple options)
10	How long do you usually use steroids? (multiple options)
	Adjuvants in General
11	Have you been using any adjuvant sepsis treatments in the last year (steroids excluded)?
11a	Why are you not using any adjuvant sepsis treatments?
12	Which adjuvant therapies have you used in your clinical practice on the ICU in the last year (outside of a trial)?
13	In which scenarios are you using IVIG?
14	In which scenarios are you using protein C?
15	In which scenarios are you using vitamin C?
16	What is your transfusion threshold for ATIII in sepsis (put in “100” if you are not measuring it before)?
	Vasoactive and inotropic drugs
17	Which VASOACTIVE/INOTROPIC substances (besides norepinephrine, dobutamine, vasopressin and epinephrine) have you used as an adjuvant rescue strategy in refractory septic shock patients in the last year?
17a	Why are you not using adjuvant vasoactive/inotropic substances?
18	Out of your last 100 patients with septic shock, how often have you used angiotensin II?A: 0–100
19	Out of your last 100 patients with septic shock, how often have you used Methylene blue? 0–100
20	Out of your last 100 patients with septic shock, how often have you used vasopressin? 0–100
21	Out of your last 100 patients with septic shock, how often did you use beta-blockers? 0–100
22	What could be a rationale to add beta-blockers to the treatment in septic shock?
	Extracorporeal blood purification
23	Have you used extracorporeal blood purification in refractory septic shock in the last year?
23a	Why are you not using extracorporeal blood purification?
24	Out of your last 100 patients with septic shock, how often have you used blood purification to stabilize a patient?
25	Which technology of blood purification have you used in your ICU in the last year and how often?
	Immunomodulators
26	Have you used intravenous Immunoglobulins (IVIG) in your ICU as adjunctive treatment in patients with sepsis in the last year?
26a	Why are you not using IVIG in septic shock patients?
27	Which IVIG preparation do you usually use for patients with sepsis or septic shock? (multiple options)
28	Do you measure IgG or IgM levels before considering IVIG treatment in sepsis patients?
29	Have you used granulocyte/monocyte colony-stimulating factor (GM-CSF) as sepsis adjunctive therapy in the last year?
29a	Why are you not using GM-CSF in septic shock patients?
30	In your clinical practice on the ICU do you use any additional adjunctive sepsis treatment that was not mentioned so far?

### Survey development

Survey questions were developed and selected by members of the Sepsis and Infection Section (SIS), all of whom are experts in the field. Question selection was based on the perceived relevance of the topics, current clinical practice, available literature, and ongoing clinical trials, with the aim of addressing areas of uncertainty and variability in the use of adjunctive therapies.

### Data collection and survey closure

The survey remained open until no additional responses were recorded for a continuous period of 14 days, at which point data collection was closed. No incentives were offered for participation.

### Data handling and analysis

All responses were collected anonymously and analyzed descriptively. Results are presented as percentages of the total number of respondents for each question. No imputation of missing data was performed. Graphical representation of the results was generated using GraphPad Prism (Version 10.0; GraphPad Software, La Jolla, CA, USA), primarily in the form of pie charts.

## Results

### Participants

A total of 442 physicians participated in this survey. Questions of this survey are listed in Table 1. 243 (55%) were male and about half of them had professional experience in the ICU for more than 10 years. Fifty-two % referred to “Intensive Care Medicine” as their primary specialty and 45% worked in a university hospital setting. Detailed demographics of the participants are summarized in Fig. 1.

### Corticosteroids

**Fig. 2:**
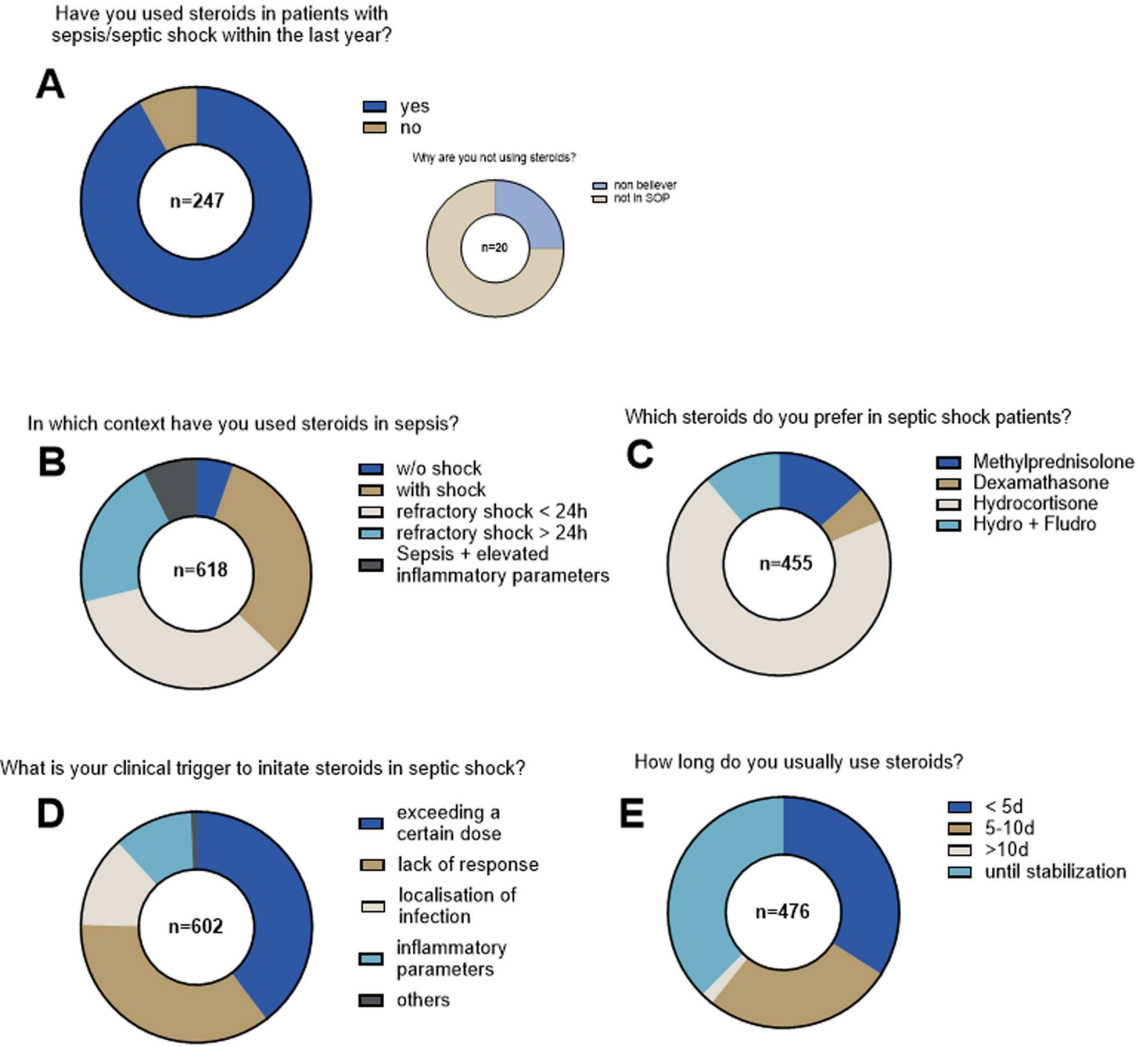
Use of corticosteroids

Given that corticosteroids are recommended by most guidelines, they are per definition not adjunctive treatments (yet they also target the host immune response). In line, only 20 out of 247 respondents (8.1%) stated that they have not used corticosteroids as sepsis adjunctive within the last year (Fig. 2A). The clinical context of their use in the remaining 91.9% respondents was predominately limited to patients in septic shock (Fig. 2B). The majority (70.1%) used hydrocortisone (HC) with a few physicians preferring the combination of HC + fludrocortisone (11.2%), methylprednisolone (13.4%) or dexamethasone (5.3%). Of note, no one stated to use fludrocortisone alone (Fig. 2C). Almost all respondents seem to have personal triggers to initiate corticosteroids ranging from severity of shock, unresponsiveness to standard treatment, site of infection up to certain levels of biomarkers such as C-reactive protein. Treatment duration was similarly heterogeneous with one-third > 5 days, one-third 5–10 days and one-third until stabilization. Very few respondents used corticosteroids for longer than 10 days (Fig. 2D,E).

### Adjunctive sepsis treatments

**Fig. 3:**
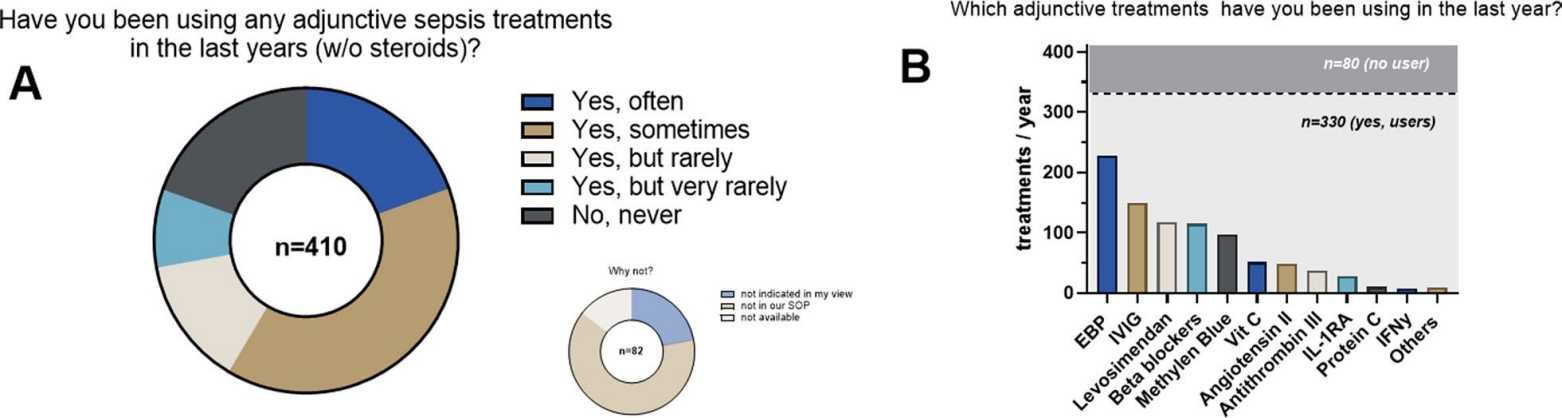
Adjunctive sepsis therapies in general (w/o steroids)

Apart from steroids, 80.5% of respondents did use adjunctive sepsis therapies in the last year; that means that 19.5% (*n* = 80/410) did not use additional adjunctive therapies and 12 of these 80 responders (15%) declared that the underlying reason was lack of availability in their institutions. The remaining 330 respondents (80.5%) used adjunctive treatments but at different frequencies, with the majority (39%) answering as “sometimes” and very different preferences. The top three answers were extracorporeal blood purification (EBP) 228/410 (55.6%); intravenous immunoglobulins (IVIG) 149/410 (36.3%); and levosimendan 117/410 (28.5%) (Fig. 3). Vitamin C is of particular interest as it has been shown in the meantime that it might be associated with an increase in mortality. The specific scenarios of its use are further summarized in Table 2.

**Table 2:** Vitamin C indications

Vitamin C indications	N (40)
All septic shock patients	27
Refractory shock	5
Capillary leakage	2
COVID	4
Malnutrition	1
Burns	1

### Vasoactive and inotropic drugs

**Fig. 4:**
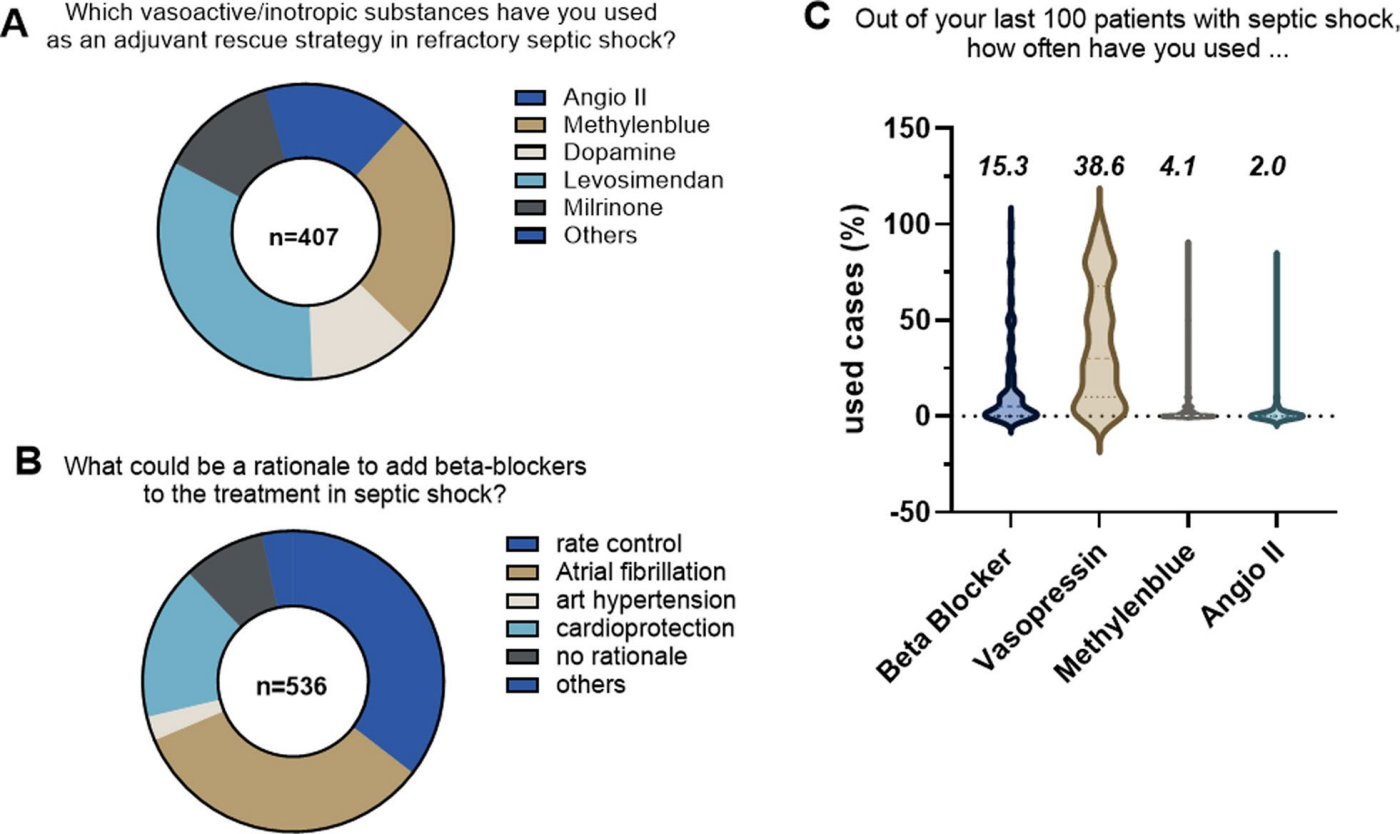
Use of vasoactive and inotropic drugs

Aside from guideline-recommended drugs like norepinephrine, vasopressin, and dobutamine, 92% of participants declared that they have used other vasoactive/inotropic agents as adjunctive rescue treatments in septic shock. In order of their quotation were mentioned: levosimendan (136/407; 33.4%), methylene blue (104/407; 25.6%), milrinone (52/407; 12.8%) followed by angiotensin II, dopamine, and others (Fig. 4A). Beta-blockers were used by 115/442 physicians (26%), mostly for rate control and atrial fibrillation. Regarding the general rationale for beta-blockers, the respondents also mentioned blood pressure management and cardioprotection (Fig. 4B). Out of the last 100 septic patients: a) beta-blockers were used on average in 15/100 patients (331 respondents); b) vasopressin was used in 39/100 patients (266 respondents); c) methylene blue was used in 4/100 patients (244 respondents); and d) angiotensin II was used in 2/100 patients (242 respondents) (Fig. 4C).

### Extracorporeal blood purification (EBP)

**Fig. 5:**
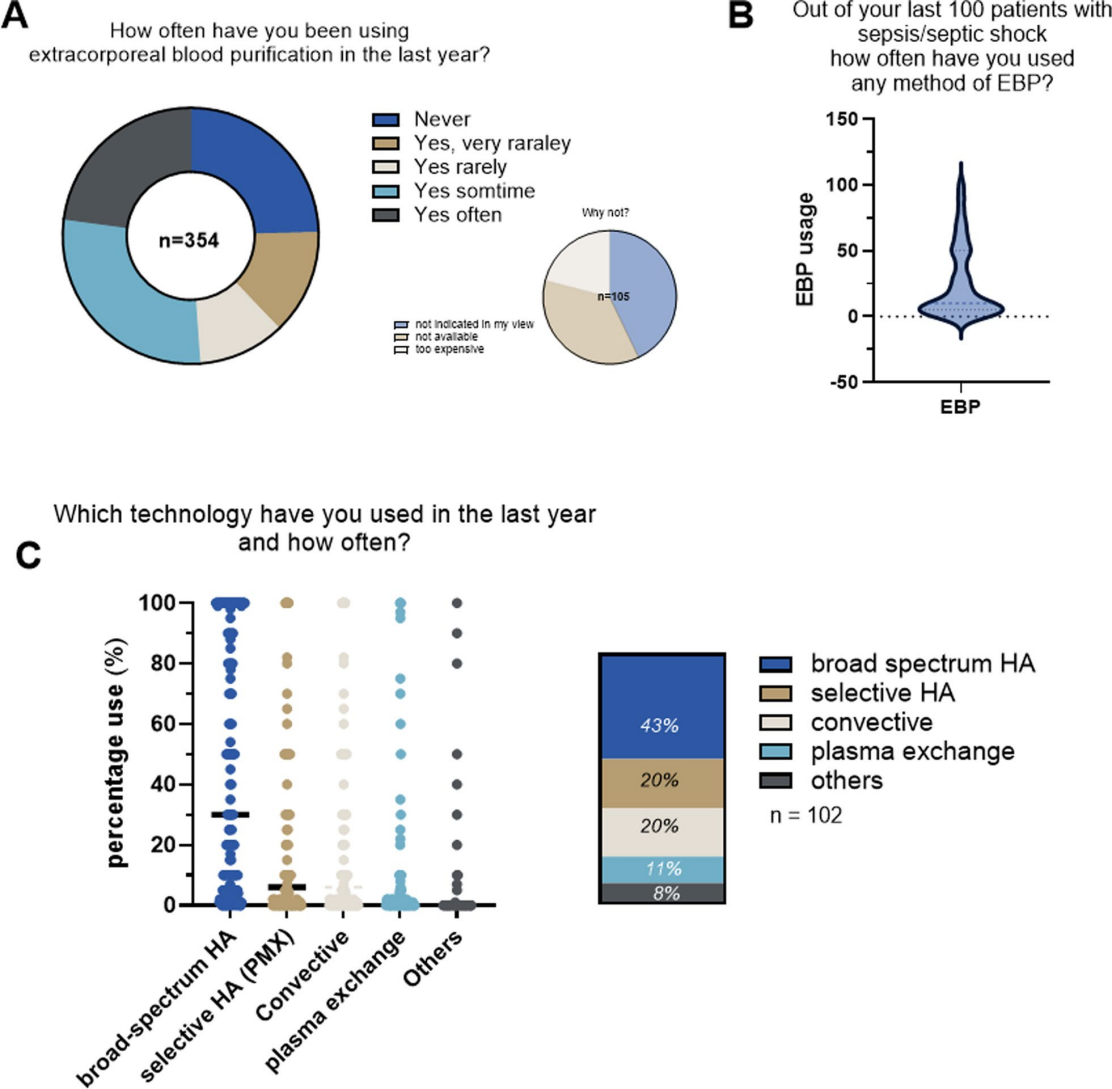
Use of extracorporeal blood purification

About three-quarters of respondents stated to use EBP technologies, while 24.6% of those who never used them acknowledged the barrier of costs (Fig. 5A). When asked in more detail regarding frequencies of EBP application, 247 respondents answered that they have used it in 26 out of their last 100 patients with refractory septic shock (Fig. 5B). The technology most frequently used was broad-spectrum hemoadsorption (43%), followed by selective endotoxin adsorption with polymyxin B (PMX) (20%), convective hemofiltration (20%), and therapeutic plasma exchange (8%, Fig. 5C). The group of other EBP is summarized in Table 3.

**Table 3:** Other extracorporeal blood purification technologies

Other EBP	N (11)
Oxiris	3
HCO	1
Septex	2
CVVHD	1
Efferon	1
Seraph	3

### Immunomodulators

Half of 345 respondents used IVIG as sepsis adjunctive in their bedside practice. Most respondents who are not using IVIG stated that they are either not in their standard of practice or simply too expensive (Fig. 5A). Of those who used IVIG to treat sepsis/septic shock, 56.4% measured IgG or IgM levels in their patients before to stratify/tailor IVIG use (Fig. 5B). IgG deficiency followed by general immunosuppression were the most common scenarios in which IVIGs were used (Table 4). Along the same line, we asked the usage of granulocyte–macrophage colony-stimulating factor (GM-CSF). With 32% of replies, GM-CSF was less frequently used than most other adjunctive therapies (Fig. 5C). Pharmacological IL-1 receptor blockade (anakinra®) was used by 27 of the 442 respondents (6.1%, Fig. 3B).

**Table 4:** IVIG indications

IVIG indication	N (124)	Percentage
Immunosuppression	21	16.9
IgG deficiency	24	19.4
IgM deficiency	13	10.5
B cell depletion	1	0.8
Hypogammaglobulinemia	2	1.6
Neutropenia	4	3.2
Autoimmunity	1	0.8
After transplantation	2	1.6
Hemato-oncological pathology	1	0.8
Toxic shock	14	11.3
Necrotizing fasciitis	14	11.3
Soft tissue infection	2	1.6
Purpura fulminans	1	0.8
Pneumococcal shock	2	1.6
Hyperinflammation/toxins	3	2.4
Multi-drug resistancy	1	0.8
Gram neg-. Abd. Infections	2	1.6
refractory shock	12	9.7
To-Piro score	4	3.2

## Discussion

This multinational survey provides a contemporary snapshot of the real-world use of corticosteroids, vasopressors, and adjunctive therapies for sepsis across European intensive care units. The principal finding is that steroids, vasopressors, and adjunctive therapies are widely used despite limited high-quality evidence supporting most interventions. Only one in five respondents reported not using any adjunctive therapy in the preceding year, indicating that intensivists frequently seek additional strategies when standard sepsis care is perceived as insufficient. Across all domains, practice was characterized by marked heterogeneity in indications, timing, duration, and patient selection, reflecting persistent uncertainty and the absence of clear, evidence-based guidance.

Consistent with international guidelines, corticosteroids remain the most widely applied intervention, [4]. The decision to initiate therapy was largely individualized, based on shock severity, hemodynamic instability, infection site, or biomarker levels. Duration of therapy varied significantly, reflecting the ongoing uncertainty regarding optimal treatment length, a phenomenon also noted in previous studies. Our question about personal preferences may be biased due to environmental factors that could influence them.

EBP technologies [12] were used by approximately three-quarters of respondents, mostly in patients with refractory septic shock. These findings underscore the continued interest in EBP as a potential strategy to mitigate the host’s dysregulated inflammatory response. Despite a low level of evidence, the reasons explaining the use of EBP are complex but might incorporate aspects of biological plausibility as well as the intensivists’ close relation to extracorporeal and technical approaches. Nevertheless, a similar percentage of EBP use in sepsis was reported in another survey specifically focused on the topic of EBP [11]. The underlying reasons may include factors such as biological plausibility and the influence of key opinion leaders in certain countries who also endorsed the survey. Broad-spectrum hemoadsorption was the preferred modality, followed by polymyxin B endotoxin adsorption, convective hemofiltration, and therapeutic plasma exchange. Cost considerations were a significant barrier for non-users, emphasizing the need for economic as well as clinical evaluation in future studies.

IVIG [13] were employed by roughly one-third of respondents, primarily in patients with documented immunoglobulin deficiencies or severe immunosuppression. Notably, more than half of users measured IgG or IgM levels prior to administration, suggesting a trend toward individualized, biomarker-guided therapy. Other immunomodulators, including GM-CSF and pharmacological IL-1 receptor blockade, were used less frequently, likely reflecting both limited availability and the relative scarcity of supporting evidence. Of note, the effect of IL-1 blockade was neutral in an earlier phase III trial that investigated a broader range of patients without biomarker stratification [14]. A post hoc analysis, however, later showed a signal toward efficacy in patients with macrophage activation features [15]. The low number of IL-1RA users should be interpreted with caution since the survey was conducted before the recent release of the *ImmunoSep* trial in 2025 [9]. ImmunoSep assessed a personalized immunotherapy, including IL-1 blockade in hyperinflamed septic patients and showed an improvement of the SOFA score by 1.4 points in the intervention group in patients with hyperferritinemia.

Vasoactive and inotropic agents beyond standard therapy were also quite commonly including levosimendan, methylene blue, and beta-blockers. Their use was often guided by hemodynamic considerations and refractory shock, illustrating the ongoing search for strategies to optimize cardiac output and vascular tone in the most critically ill patients. The continued use of levosimendan in septic shock is surprising given the lack of benefit observed in the LeoPARDS randomized trial [16]. Similarly, the continued use of beta-blockers despite the recent results of the STRESS-L [17] and Landi-SEP [18] RCTs, which investigated the use of landiolol in septic shock and was stopped prematurely due to lack of benefit and possible harm.

Overall, our survey highlights substantial heterogeneity in both the availability and use of adjunctive sepsis therapies. This variability likely reflects differences in local protocols, resource availability, clinician experience, and interpretation of the existing, often limited evidence base. Nevertheless, systematic collection and rigorous analysis may help identify patterns of use, patient characteristics, and clinical outcomes across diverse settings. Such data can provide valuable insights into the effectiveness, safety, and appropriateness of adjunctive therapies in routine practice, complementing evidence from randomized controlled trials. At the same time, it underscores a critical need for well-designed, multicenter RCTs to clarify the role of these interventions and to provide guidance for individualized therapy.

This study has several limitations. First, it relies on self-reported data, which may be affected by recall and reporting bias. Second, dissemination through the ESICM newsletter and co-author professional networks may have introduced selection bias, potentially over-representing clinicians with a specific interest or experience in adjunctive sepsis therapies. Third, the varying levels of physician training could result in overinterpretation and limit the transferability of findings. However, this very heterogeneity reflects the real-life treatment of septic patients. Fourth, the survey design did not allow assessment of patient-level outcomes, precluding any conclusions regarding the effectiveness or safety of the reported interventions. In addition, because the survey was distributed through open channels, a clear denominator was not available and a formal response rate could not be calculated, limiting interpretation of the representativeness of the findings.

In conclusion, this survey demonstrates that besides steroids, adjunctive therapies are deeply embedded in contemporary sepsis management across European ICUs, despite the absence of robust evidence for most interventions. EBP and IVIG emerged as the most used strategies, typically reserved for severe or refractory cases and often guided by clinician judgment or selected biomarkers rather than standardized protocols. The substantial variability observed in availability, indications, and patterns of use underscores both the clinical relevance of these therapies and the profound uncertainty surrounding their benefit. These findings highlight a critical gap between guideline recommendations and bedside practice and reinforce the urgent need for well-designed, adequately powered, multicenter randomized trials. Future research should focus on identifying responsive patient subgroups, defining optimal timing and stopping rules, and integrating precision-medicine approaches to move adjunctive sepsis therapy from empiricism toward evidence-based individualization.

## Data Availability

All data are available with and can be shared upon request to the corresponding author.
